# Case Report: Narrative therapy combined with Satir model for the treatment of trichotillomania

**DOI:** 10.3389/fpsyt.2025.1588939

**Published:** 2025-09-02

**Authors:** Haiyan Liu, Xiuxiu Chen, Xiaochao Wan, Dan Wang, Zhiguo Chen

**Affiliations:** Department of Psychiatry, 82nd Group Army Hospital of People’s Liberation Army (PLA), Baoding, Hebei, China

**Keywords:** trichotillomania, integrative psychotherapy, narrative therapy, Satir model, case report

## Abstract

**Background:**

Trichotillomania is a chronic psychiatric and behavioral disorder characterized by recurrent hair-pulling, often leading to significant distress and impairment. Long-term psychotherapeutic case reports remain scarce, especially those integrating Narrative Therapy and the Satir Model in culturally-specific and resource-limited contexts.

**Case Presentation:**

We present a two-year integrated psychotherapy case for a 28-year-old woman with trichotillomania, utilizing Narrative Therapy and Satir Transformational Systemic Therapy. Assessments included the Massachusetts General Hospital Hair Pulling Scale (MGH-HPS), Self-Rating Anxiety Scale (SAS), Personal and Social Performance Scale (PSP), and Clinical Global Impression (CGI). Hair-pulling episodes decreased from 150 per day to 5, and MGH-HPS, SAS, and PSP scores improved substantially from baseline to end of treatment and one-year follow-up. The patient maintained gains post-treatment, with improved self-worth and social functioning.

**Conclusions:**

This case supports the clinical utility of a Narrative Therapy–Satir Model integration for trichotillomania. The flexible, person-centered approach yielded lasting symptom and functional gains. Limitations include reliance on self-report and potential bias.

## Introduction

Trichotillomania, also known as hair-pulling disorder, is a chronic and often debilitating psychiatric condition characterized by recurrent, irresistible urges to pull out one’s hair, resulting in noticeable hair loss and significant distress or functional impairment (1). First described by Hallopeau in 1889 (2), trichotillomania is estimated to affect approximately 1–2% of the general population, with a higher prevalence in females. The disorder frequently begins in childhood or adolescence and is often concealed by affected individuals due to feelings of shame or embarrassment, contributing to delays in seeking treatment and increased psychosocial burden (3).

Standard treatments for trichotillomania include pharmacotherapy and behavioral interventions, particularly habit reversal training and cognitive-behavioral therapy (4). However, many patients experience only partial symptom relief or may be reluctant to engage in conventional treatments, especially in culturally-specific or resource-limited settings. There is growing recognition that innovative, integrative psychotherapeutic approaches are needed to address the complex emotional, relational, and identity issues underlying chronic hair-pulling.

Narrative Therapy, which emphasizes externalizing the problem and reconstructing personal meaning through storytelling, has shown promise in empowering individuals to re-author their experiences and identities (5), The Satir Model, a systemic and experiential approach, focuses on enhancing self-worth, improving family dynamics, and fostering emotional integration (6). despite the theoretical compatibility of these approaches, empirical reports documenting their long-term, combined application for trichotillomania remain rare, particularly in East Asian and low-resource contexts.

This case report follows CARE guidelines and aims to: (1) clarify the theoretical rationale for integrating Narrative Therapy and Satir Model interventions; (2) describe outcome measures and analytic methods; (3) present a visual and narrative timeline of the treatment process; and (4) include the patient’s perspective on recovery.

## Patient information

### Demographics

28-year-old married woman from Baoding, China.

### Presentation

Sought psychiatric care before her wedding, reporting severe, chronic hair-pulling since age 8.

### Family history

No formal psychiatric diagnoses reported, but her mother exhibited hair-pulling in childhood, which resolved spontaneously in adulthood.

### Social history

Grew up in a dysfunctional family (paternal illness, parental conflict), assumed significant family responsibilities from a young age.

The key events, interventions, and clinical outcome measures of this patient’s treatment are summarized in **Table 1**.

**Table 1:** Timeline.

Date/phase	Key Event/treatment	Session frequency	MGH-HPS	SAS	PSP	CGI-SI	CGI-GI	CGI-EI
Baseline (0 mo)	Assessment	—	28	68	60	6	—	—
Phase 1 (0–6 mo)	Alliance, Externalization, Genogram	1/week (6 mo)	↓					
Phase 2 (7–16 mo)	Iceberg, Unique Outcomes, Reauthoring	1/week (10 mo)	↓					
Phase 3 (17–24 mo)	Family Sculpture, Role-play, Internal resources	1/week (8 mo)	3	30	85	2	1	3
1-year Follow-up	No formal sessions	—	3	30	85	2	1	3

## Diagnostic assessment

- DSM-5 Criteria: Meets criteria for trichotillomania (1).
- MGH-HPS: Seven items, 0–28, higher=more severe (7).
- SAS: 20 items, 25–100, >63 = moderate to severe anxiety (8).
- PSP: 0–100, higher = better function (9).
- CGI: SI (Severity, 1–7), GI (Global Improvement, 1–7), EI (Efficacy Index) (10).
- Statistical Analysis: Paired t-test compared baseline and 24-month outcomes. Cohen’s d computed for effect size.

## Therapeutic intervention

### Theoretical framework

Intervention was not grounded in formal psychodynamic psychotherapy. Rather, it was an integrative, person-centered approach, combining Narrative Therapy (externalization, re-authoring, unique outcomes) and the Satir Model (iceberg metaphor, family sculpting, self-worth enhancement). Psychoanalytic concepts (e.g., defense mechanisms, unconscious conflict) were used only for explanatory context.

### Satir model in practice

The Satir Model, a systemic and experiential family therapy, aims to increase congruence, self-esteem, and family harmony by exploring communication stances, family roles, and emotional needs (6, 11). In this case, key techniques included the “iceberg metaphor” (exploring visible behavior vs. underlying emotions/needs) and family sculpting (mapping family dynamics).

### Intervention phases

The integrated psychological intervention process for this patient is outlined in **Table 2**.

**Table 2:** Integrated psychological intervention process for trichotillomania.

Phase	Treatment objective	Treatment content and key techniques	Frequency & duration	Reflections on treatment process
Phase 1	Establish a therapeutic alliance and explore the deep meaning of symptoms and their impact on the patient.	① Establish a therapeutic relationship: sincerity, empathy, respect, listening, avoiding judgment. ② Narrative externalization techniques: naming (“the fool”), observer perspective (“What do you notice? Good? Bad?”); relationship repositioning. ③ Drawing a family genogram: clarify roles, relationship mapping (emotional direction), find trauma sources; psychoeducation. ④ Reconstruct the narrative meaning of symptoms: select key events-multiple perspectives-third-person retelling-emotional re-experience.	Once a week, total of 6 months	① Emotional release and relief, anxiety reduced; ② Externalization: shift the focus, understand the external meaning; ③ Sense of security from therapeutic alliance; relaxation techniques, support provided.
Phase 2	Deeply explore the underlying emotional needs and beliefs behind symptoms, strengthen self-identity, and reconstruct a positive self-narrative.	① Iceberg metaphor: draw iceberg (behavior/emotional needs); explore iceberg elements. ② Narrative “unique outcomes” technique: search for positive exceptions, re-authoring, reconstruct narrative. ③ Select key events: connect them, extract their meaning, participate and witness.	Once a week, total of 10 months	① Gradual recognition and differentiation of emotional needs; ② Shift from symptom to internal emotional needs; ③ Finding and receiving affirmation from key events, reconstructing self-identity, and emotional resonance.
Phase 3	Restore social functioning, build adaptive coping strategies, consolidate new self-narrative, and promote the generalization of positive behavioral patterns.	① Family sculpture: map relationships, clarify positions (distance and closeness), highlight communication structure. ② Role-play: simulate real-life communication, practice expressing needs and emotions, practice refusal, and assertion. ③ Build internal resources: mindfulness meditation, self-affirmation journal, emotion regulation skills, replace hair-pulling with adaptive behaviors; farewell letter writing, ritualized closure.	Once a week, total of 8 months	① Better understanding of family and self in context; ② Enhanced self-worth and belief in being loved; ③ Social functioning restored and stabilized; sense of wholeness and integration, preparation for transition.

#### Phase 1: Symptom deconstruction (0–6 months)

- Establishment of alliance; externalization of hair-pulling (“Big Fool”).
- Genogram construction; trauma mapping; psychoeducation.

#### Phase 2: Cognitive/emotional reconstruction (7–16 months)

- Iceberg metaphor to connect behavior and emotional needs.
- Narrative “unique outcomes” and “re-authoring” to reconstruct self-identity.

#### Phase 3: Relationship remolding and social function restoration (17–24 months)

- Family sculpting and role-play to foster healthy communication.
- Mindfulness, self-affirmation journaling, ritual closure (farewell letter).

## Results

### Quantitative outcomes

Key assessment scores at baseline, mid-treatment, end of treatment, and follow-up are presented in **Table 3**.

**Table 3:** Assessment scores at key timepoints.

Assessment index	Baseline	Mid-treatment (12 mo)	End of treatment (24 mo)	1-Year follow-up
MGH-HPS	28	9	3	3
SAS	68	32	30	30
PSP	60	80	85	85
CGI-SI	6	3	2	2
CGI-GI	—	3	1	1
CGI-EI	—	—	3	3

### Statistical analysis

A paired samples t-test comparing baseline and post-treatment (24 months) MGH-HPS scores showed a significant reduction: *t*(1) = 7.07, *p* <.05; Cohen’s *d* = 2.65 (large effect size).

### Visual timeline

**Figure 1:**
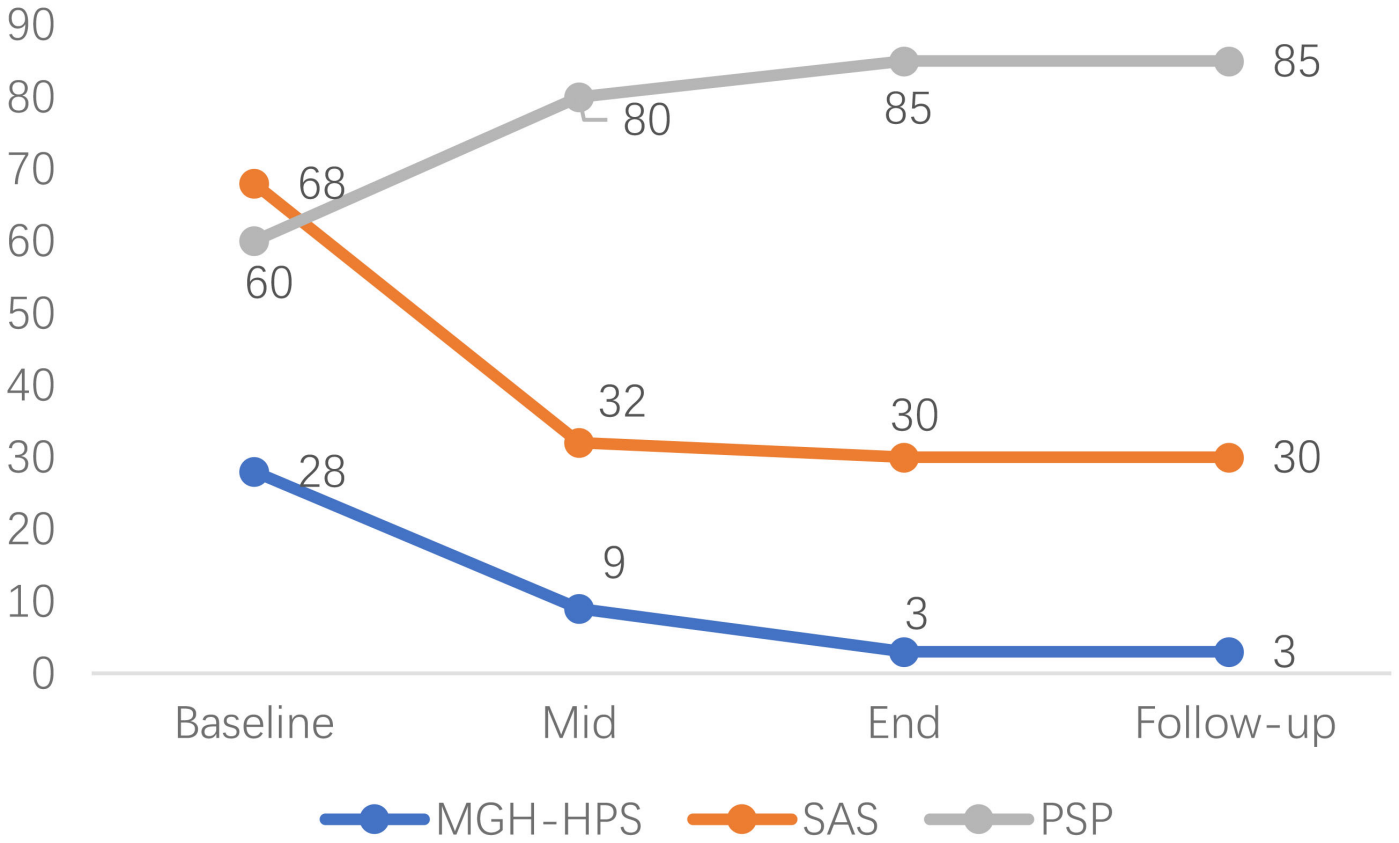
Visual timeline of key assessment scores (MGH-HPS, SAS, PSP) across treatment phases and follow-up.

A visual timeline of key assessment scores across treatment phases and follow-up is displayed in **Figure 1**.

### Qualitative/functional outcomes

- Hair-pulling episodes reduced from 150/day to 5/day; normalized scalp appearance.
- Anxiety (SAS) decreased from severe (68) to mild (30).
- Social functioning (PSP) improved from impaired (60) to normal (85); secured stable employment; passed teacher qualification examination.
- Maintained gains at 1-year follow-up; rare, controlled relapses not affecting daily appearance or function.

### Patient perspective

“Before therapy, I felt hopeless and ashamed. I always hid my bald patches with wigs, afraid of being found out. Over time, I understood my hair-pulling was not just a bad habit but a way I coped with pain and anxiety. Naming my problem and talking about my family helped me accept myself. Now, I can go out without a wig and am not ashamed. I believe I am capable and worthy, and I have plans for my future. Even when I feel the urge, I can control it.”

## Discussion

This case illustrates the potential of integrating Narrative Therapy and the Satir Model for chronic trichotillomania. Despite the patient’s initial rejection of medication and cognitive restructuring therapy (12), an individualized, flexible approach achieved remission of hair-pulling symptoms, restored social function, and enhanced self-worth.

Theoretical integration: This intervention was guided by an integrative, not strictly psychodynamic, approach (13). Narrative Therapy and the Satir Model jointly addressed cognitive, emotional, and relational aspects, supporting sustained change, especially in culturally specific and resource-limited contexts.

Scales: The MGH-HPS, SAS, PSP, and CGI provided standardized, repeated measures of symptom severity, anxiety, and functioning. However, all were self-reported, and the patient’s desire to please the therapist may have introduced social desirability bias, especially in latter assessments.

Behavioral drives: While OCD and trichotillomania are often compared, their motivational drives differ: OCD behaviors are primarily anxiety-reducing, while hair-pulling can be both tension-reducing and pleasure-driven. In this case, initial relief from anxiety was reported, but over time, the behavior became a more complex means of emotional regulation and seeking control.

### Limitations

- Single-case design: Generalizability is limited.
- Therapist bias: Strong alliance may have influenced patient responses and therapist ratings.
- Self-report instruments: Lack of third-party corroboration and potential for response distortion due to social desirability.
- No session fidelity monitoring: Treatment integrity was not formally measured.

Future directions: Larger, controlled studies with independent assessment and session fidelity are warranted. Exploring cultural adaptation and optimizing integrative protocols may improve accessibility and outcomes.

## Conclusion

Integrative psychotherapy combining Narrative Therapy and the Satir Model may offer a valuable option for trichotillomania, especially when conventional approaches are declined. This case highlights the importance of person-centered, flexible, cross-theoretical interventions and the need for rigorous outcome monitoring and reporting in future research.

## Data Availability

The original contributions presented in the study are included in the article/supplementary material. Further inquiries can be directed to the corresponding author.
